# A Corpus-Based Investigation of Extra-Textual, Connective, and Emphasizing Additions in English-Chinese Conference Interpreting

**DOI:** 10.3389/fpsyg.2022.847735

**Published:** 2022-05-30

**Authors:** Ruitian Li, Andrew K. F. Cheung, Kanglong Liu

**Affiliations:** Department of Chinese and Bilingual Studies, Hong Kong Polytechnic University, Hong Kong, Hong Kong SAR, China

**Keywords:** consecutive interpreting, interpreting additions, visibility, discourse markers, press conference

## Abstract

Findings from conference interpreting research in the Chinese context have suggested that interpreters barely produce extra-textual additions in rigidly structured press conferences, and that adding connectives and intensifiers is only required to help the English-speaking audience capture the logic embedded in implicit Chinese interclausal relations. Previous research in the Chinese context has tended to draw data almost exclusively from the Chinese Premier's Press Conference interpreting, which features interpreting from Chinese into English. In order to enrich conference interpreting corpora in Asia and to examine additions in the opposite interpreting direction for the same language pair, this study drew on authentic materials of six interpreted press conferences held at the American Institute in Taiwan (AIT). Contrary to previous research, our results showed that conference interpreters exhibited a certain degree of “visibility” through producing extra-textual additions, which is typical of interpreting in various community-based settings. Moreover, the addition of extra connectives and intensifiers that are common in Chinese-to-English interpreting was also identified. It is proposed that the interpreters' production of extra-textual additions is connected with the specific context of the AIT, whereas the connective and emphasizing additions are to a large extent caused by the grammaticalization process wherein particular linguistic devices change into discourse markers to fulfill the communicative needs in both English and Chinese.

## Introduction

The purpose of this corpus-based study is to report on the primary types of additions identified in authentic English-to-Chinese interpreting tasks carried out by professional interpreters. The research data was drawn from the American Institute in Taiwan (AIT) Interpreting Corpus. Since the AIT functions as a de-facto US embassy in Taiwan, its interpreting activities are oriented around informing the Taiwanese audience of the US policies on Taiwan and because of this, it represents high-profile conference interpreting in Taiwan. The constructed AIT Interpreting Corpus is also a response to (Bendazzoli's, 2018) call to enrich conference interpreting corpora in Asia, as previous studies have primarily sourced information from the same data (i.e., the Chinese Premier's Press Conference (CPPC) interpreting). However, press conferences hosted by Chinese Premiers tend to be heavily scripted, and so interpreters at these conferences may have prepared, well in advance, for translating the content in order to showcase the ruling party's achievements (Zhang et al., 2002). Therefore, studies based on this type of corpus may not reflect situations in which interpreters do not have as much access to the speech information as those working for a Chinese Premier.

With respect to addition, earlier studies of interpreting have tended to alert readers to its potential for inducing translation errors (Barik, 1971; Altman, 1994). Since then, researchers have tended to view additions as a strategy to clarify the information that is assumed not to be conveyed clearly by the original speaker (e.g., Kalina, 1998; Donato, 2003; Bartlomiejczyk, 2006; Liontou, 2011). Interpreting strategies are “methods that are potentially conducive to solving particular problems encountered by interpreters or generally facilitating the interpreter's task and preventing potential problems” (Bartlomiejczyk, 2006, p. 152). However, without relevant introspective data (Ericsson and Simon, 1993) from both the source speaker and the interpreter, it would be rather risky to associate additions with problem-solving on the part of the interpreter. For this reason, the additions in this study will not be treated as problem-oriented strategies, but as linguistic devices or verbal utterances added independently by the interpreters in the target text.

Taking a corpus-based approach, this study aims to examine the primary types of additions identified in the dataset and their ensuing implications on the interpreted discourse. In addition, since interpreting is “a united but contextually-situated practice” (Downie, 2021, p. 339), this study reflects on the various factors, both specific to and beyond the given interpreting setting at the AIT press conference, that may condition or predict the occurrence of the identified types of additions in English-Chinese interpreting.

## Typology of Additions

Table 1 shows a typology of additions on which the present study is based. This typology was based on a systematic review of related literature and its applicability in the Chinese language. We have identified three types of additions in the American Institute in Taiwan (AIT) press conference interpreting, and have given these the labels A1, A2, and A3: extra-textual, connective and emphasizing, respectively.

**Table 1:** Typology of additions for the present study.

**Category**	**Definition**	**Example**
Extra-textual additions (A1)	Interpreters' utterances that have no corresponding parts in the source utterances.	•Text-oriented: requests for clarification (also clarify for the audience by giving more relevant information); requests for time to translate; comments on one's translations, etc. •Interaction-oriented: requests to observe turn-taking order; invitations to start, stop, or continue talking; requests for solicited but not yet provided information, etc.
Connective additions (A2)	Additions serving to connect or link together utterances or parts of utterances.	Adding connectives such as *because, so, in addition, although, but, if…then, apart from, not only…but also, first, second, third, with respect to*, etc.
Emphasizing additions (A3)	Additions serving to emphasize or increase the force of the utterance or part of the utterance in the source text.	Adding intensifiers such as *very/ quite, actually/ in fact, certainly/ indeed, always, greatly/ considerably, continuously*, etc.

Type A1 additions are designated as extra-textual additions in this study. These are similar to (Wadensjö, 1998) “non-renditions,” which refers to interpreters' utterances that do not have a corresponding counterpart in the preceding source language and are “visibly designed to do coordinating work” (p. 109). Specifically, non-renditions can be either text-oriented, whereby interpreters confirm or clarify information, or interaction-oriented, where they organize and control turn-taking (Wadensjö, 1998; Cheung, 2017). Of particular note is that the term “non-rendition” originally established by Wadensj is potentially misleading given that it has been misused by some researchers (e.g., Takimoto and Koshiba, 2009) to denote “non-interpreting behaviour” (p. 15), that is, omission. So, to avoid confusion, this study uses the term “extra-textual addition” to refer to such interpreting behavior.

By contrast, type A2 and A3 additions draw on the categorization of Jacobsen (2002), which provides a systematic analysis of court interpreters' additions. Certain additions serve “to connect, or link together, utterances or parts of utterances” and “to provide clarity and orderliness” for the listeners (Jacobsen, 2002, p. 179). These are Connective additions, labeled A2 in this study. Jacobsen's emphasizing additions serve “to emphasize, or increase the force of, the utterance, or part of the utterance in the source text” (ibid., p. 186), and these are denoted by A3 for this study.

## Literature Review and Theories

### Extra-Textual Additions (A1) and Interpreters' Visibility

Translation and coordination are two inseparable aspects pertinent to the role of interpreters (Wadensjö, 1998). In contrast to “translation” which is to relay an original message, “coordination” carried out by interpreters is reflected in “extra-textual additions,” i.e., utterances produced on interpreters' own initiative for either textual clarification or interactional management (ibid.). Studies investigating interpreters' extra-textual additions used to draw data from community-based settings featuring dialog interpreting. The findings, as such, helped to deconstruct the idea that interpreters simply act as “conduits,” and attested to their active participation in co-constructing the target discourse.

In interpreter-mediated legal, medical, and educational encounters, interpreters may initiate extra-textual additions toward either side of the institutional participants, whether defendants or judges (e.g., Hale, 2004; Gallez and Maryns, 2014; Braun, 2017; Cheung, 2017, 2018; Arumí and Vargas-Urpi, 2018), patients or doctors (Merlini and Favaron, 2005; Baraldi and Gavioli, 2007; Baraldi, 2012), or parents or teachers (Davitti, 2013; Arumí and Vargas-Urpi, 2017). Researchers' attitudes toward extra-textual additions are relatively mixed, with some indicating their contribution to facilitating interactional goals, while others expressing concern that any side talk accompanying an interpreter's extra-textual additions (between him/her and one of the parties) can exclude other monolingual interlocutors from the communicative event.

The concept that is often discussed together with extra-textual additions is interpreters' “visibility.” It was first put forward by (Angelelli, 2004a,b) to draw attention to the tension between interpreters' real practice and professional discourse, in which interpreters are painted as quasi-invisible conduits. An example of this “invisibility” ideal can be found in the AIIC code of ethics (Association Internationale des Interprètes de Conference) that prescribe interpreters to “make them [the audience] forget they are hearing the speaker through the interpreter” (AIIC [Association Internationale des Interprètes de Conference]., 1999). In this sense, interpreters are not expected to attain their own speaking position but have theirs merged with that of the source speaker. Then by contrast, “visibility” means that the interpreter makes the audience perceive as if s/he is speaking as an independent speaker and thus becomes visible from the interpreted texts. According to Angelelli (2004a, p. 11), visibility manifests itself when the interpreter organizes turn-taking and controls the traffic of information, explains terms or concepts, filters information, and aligns with or even replaces one of the communicative parties. All of these behaviors are accompanied by interpreters' articulation of extra-textual additions (Wadensjö, 1998; Cheung, 2017).

In fact, due to the highly interactional nature of dialog interpreting, extra-textual additions for ensuring that the dialogue runs smoothly, such as organizing turn-taking and requesting for clarification, have been validated by some interpreting codes. For example, the NAJIT code (National Association of Judiciary Interpreters and Translators) stipulates: “Guessing should be avoided. Court interpreters who do not hear or understand what a speaker has said should seek clarification” (NAJIT, 2016). However, in regard to some high-profile interpreting, such as press conference interpreting in political or diplomatic settings, even extra-textual additions, as mentioned above, are not encouraged. The key reason for this is also determined by the rigidly structured format of press conferences, which creates little space for interpreters to coordinate interactions. In fact, regarding conference interpreting undertaken in the Chinese context, notably mainland China's CPPC interpreting, there is little evidence suggesting that interpreters have produced extra-textual additions of the kinds that would lead them to be perceived as “visible.”

Ozolins (2016) and Downie (2017) have debated the appropriateness of the given term and the extent to which interpreters' visibility manifested through extra-textual additions can be allowed. It is worth mentioning that the two scholars have both agreed on giving priority to the specific interpreted contexts that may affect interpreters' behaviors. That is to say, rather than arguing whether the identified extra-textual additions comply with any decontextualized professional norms, it would be more useful to focus the analysis on what interpreters actually do in each specific context. With this in mind, extra-textual additions reflecting interpreters' visibility in the present study will be discussed with recourse to the specificity of interpreting at the AIT (see Section Discussion).

### Connective and Emphasizing Additions (A2 & A3) in Chinese-to-English Conference Interpreting

Researchers working on Chinese interpreting and conference interpreting in authentic settings tend to source data from the Chinese Premier's Press Conference (CPPC), which features interpreting from Chinese to English. This line of inquiry (e.g., Cheung, 2009; Hu and Tao, 2009, 2010, 2012; Fu, 2017) examining the linguistic make-up of interpreted texts has identified two common additions: connectives and intensifiers.

One line of research prefers to take a norm-governed attitude toward the recurring patterns or regularity of shifts emerging from the interpreted texts. As has been found by Hu and Tao (2010), the use of connectives occurs with the highest frequency in interpreted English texts if compared to both translated and original English texts, thus marking a “normalization” feature of interpreted English. In a similar vein, with repeated additions of intensifiers such as “very” found before English adjectives, Hu and Tao (2012) point out a “strengthening” norm characterizing interpreted English. In other words, interpreters affiliated with the Chinese government are inclined to intensify the semantic strength of attitudes conveyed by Chinese political leaders.

Another line of research sets out from a generally strategy-oriented perspective, linking the additions under discussion to signs of the interpreter's explicitations, or to their coherence-building competence. Drawing on Halliday's (1994). Systematic Functional Grammar framework, which views language as organized around three major strands of meaning, Hu and Tao (2009) argue that additions may reflect interpreters' attempts to explicitate experiential, interpersonal, or textual meanings. Fu (2017) subsumes connectives and intensifiers under the broad heading of metadiscoursal devices (Hyland, 2005), and insists that such additions represent interpreters' strategy to maximally transfer the speaker's communicative intent for the listeners. In particular, when interpreting is carried out from Chinese to English, adding connectives is indispensable (Cheung, 2009) in facilitating the English-speaking audience in grasping the logic embedded in implicit Chinese interclausal relations.

It is important to note that these grammatical items in both English and Chinese are undergoing a process of grammaticalization (Lehmann, 1985; Hopper and Traugott, 2003), whereby they evolve from functioning as clausal linking devices to discourse makers (DMs) that signal “a sequential relationship between the current basic message and the previous discourse” (Fraser, 1990, p. 383). Despite the lack of a universally accepted definition, discourse markers are generally understood as words and phrases that serve to denote logical, spatial or temporal relationships among texts or discourse and to promote the hearers' understanding of utterances and various coherent elements within the discourse (e.g., Fraser, 1990, 1999; Carter and McCarthy, 2006).

In a similar way to the phenomenon of grammaticalization in English (Traugott, 1995), certain Chinese connectives are characterized by a shift away from semantic content toward more functional contributions. Namely, Chinese connectives, such as 因為 *yinwei* (because), 所以 *suoyi* (so), 然後 *ranhou* (then), etc., have also been found to show various degrees of grammaticalization, in a way that they contribute less to the clausal proposition but more to coherent relations between discourse units (Wang and Huang, 2006). In this way, these connectives function the same as DMs to connect conceptually-related events or actions in communicative contexts.

Besides connectives, some intensifiers have also undergone the grammaticalization process, such as *actually* and *in fact* in English (Traugott, 1995; Traugott and Dasher, 2002) and the corresponding equivalents 其實 *qishi* and 事實上 *shishishang* in Chinese (Wang et al., 2010). When used as DMs, these intensifiers will perform the function of evidentiality rather than epistemic modality, meaning that they contribute less to asserting the “truth” of a fact and more to allowing the speaker to express his/her propositional attitudes to achieve discourse coherence. Specifically, *actually* and *qishi* can serve to relate the proposition to sections of prior discourse by clarification, elaboration or even contradiction. Despite the discourse-oriented function, these intensifiers will enable the interpreter to express emphasis on upcoming information and to draw the listener's attention to various cohesive or coherent elements within the discourse.

So far, research on additions in the Chinese-English language pair has been overwhelmingly restricted to interpreting from Chinese into English. Few studies have focused on the additions of connectives and intensifiers in the context of English-to-Chinese conference interpreting. Since Chinese is a highly paratactic language in which interclausal relations tend to be inferred from the contexts instead of using connectives and intensifiers (Wang, 1984; Lian, 2010), research into English-to-Chinese interpreting can reveal some interesting patterns that might otherwise remain hidden in the other direction.

### Research Gap and Research Questions

The above literature review shows that findings on additions are closely related to interpreting settings. Specifically, previous research shows that extra-textual additions only exist in community-based interpreting settings while structured press conferences give little latitude for interpreters to utilize such additions. With regard to conference settings, few studies have examined all three types of additions, i.e., extra-textual, connective and emphasizing additions, and this is even rarer in English-to-Chinese interpreting. It is believed that this study can fulfill this research gap by examining the use of additions using a corpus comprised of English-to-Chinese interpreting data.

Accepting that interpreting is “a united but contextually-situated practice” (Downie, 2021, p. 339), this study aims to examine the interpreters' additions in interpreting activities at the American Institute in Taiwan (AIT) and seeks possible explanations behind such interpreting phenomenon. This study aims to answer two research questions:

(1) What are the frequencies of extra-textual, connective and emphasizing additions in the corpus, if they can be identified?
(2) What are the functions of these types of additions in relation to the specificity of AIT and English-to-Chinese interpreting?

Methodologically, this study adopts a descriptive corpus-based approach to the three types of additions in the interpreted product. Apart from identifying the frequency of each type of addition, the research is focused on how these additions can effect changes in the functional aspects of target language and at the level of discourse.

## Corpus and Procedures

The corpus contains six sessions of press conference spanning from the year 2006 to 2009. It is noted that the American Institute in Taiwan (AIT) may hold press conferences once or twice per year in Taiwan, as determined specifically by the US government. But due to some confidentiality concerns, these press conferences have no longer been publicly accessible since 2012. The six sessions used as data in this study were held by two American Directors, i.e., Stephen Young and William Stanton, who are also seasoned diplomats sent by the US government to inform the Taiwanese audience of the US policies about Taiwan. As a de-facto US embassy in Taiwan, the AIT plays a significant role in promoting the US-Taiwan relations through addressing various inter-social needs between the two societies. These include a wide spectrum of hot-button issues, including Taiwan's elections and international presence, American support for Taiwan's military defense, US-Taiwan cooperation, and cross-Strait relations. Of particular note is that these issues addressed in the AIT always attracts considerable media attention in Taiwan. So, given the AIT's importance toward the US-Taiwan relations and its extensive media coverage, the press conference interpreting held by such an institute can be considered representative of high-profile conference interpreting in Taiwan.

In terms of format, each press conference starts with an individual speech given by an incumbent AIT Director, which is followed by a long question-and-answer session for various on-site media outlets. The interpreting is conducted from English to Chinese in consecutive mode by female professional Taiwanese interpreters, whose first language is Mandarin Chinese and second language is English. The interpreters work for the AIT on a temporary basis, which is not unusual considering freelancing is a preferable working mode for even professional interpreters in Taiwan (Setton and Guo, 2009).

With orthographic transcriptions carried out based on the audio-video recordings available on the official website of the AIT, the data under investigation amounts to ~7 h and a half in duration, and contains 56,475 tokens in total (27,700 tokens in English and 28,775 in Chinese). The English tokens were counted based on the total words of the English ST and the Chinese tokens were based on the analyzable word tokens after tokenization (Xiao and Hu, 2015, p. 47).

In order to identify the three types of additions in question, source texts and target texts were manually segmented and aligned at the level of natural utterance units. Using the ParaConc corpus tool, intertextual comparisons between ST and TT could be conducted to detect interpreters' use of the three addition types.

To enhance the reliability of the coding process, the authors cooperated in annotating the additions used by the interpreters in the target texts. Unclear cases regarding the addition types were discussed until final agreements were reached. If the observed frequency outnumbers 100, it was normalized in terms of its occurrence every 10,000 tokens of the interpreted texts. Illustrative examples are provided to showcase how the various types of additions are worthy of scholarly attention in conference interpreting conducted from English to Chinese.

## Analysis and Results

### A1: Extra-Textual Additions

**Figure 1:**
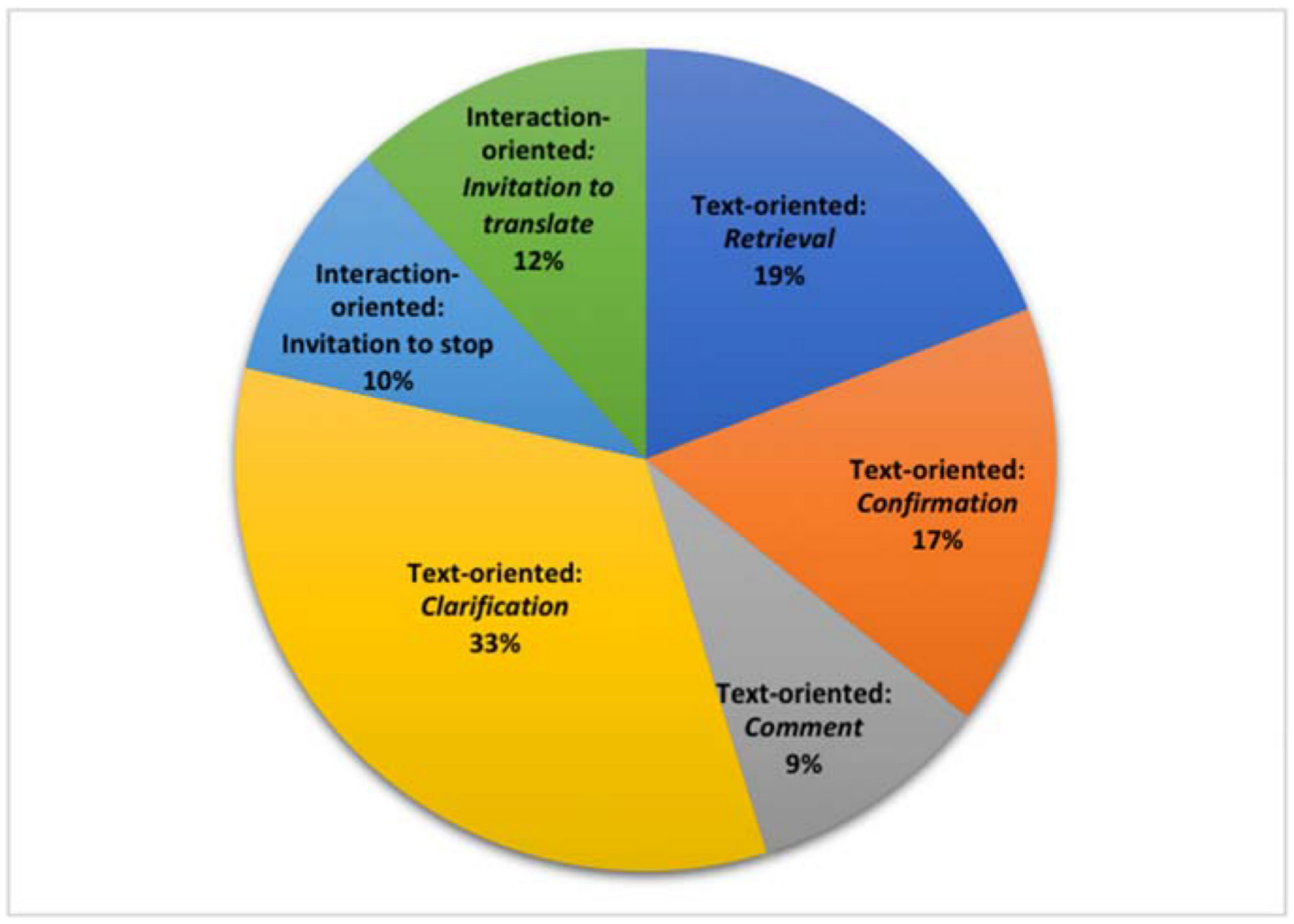
Frequency and types of extra-textual additions.

Extra-textual additions only accounted for a small proportion of additions, with only 42 occurrences in total. As shown in Figure 1, 78% of them are text-oriented while the rest 22% are interaction-oriented, among which, most are concordant with the non-rendition types that previous researchers have reported in interpreter-mediated legal and medical encounters (e.g., Wadensjö, 1998; Merlini and Favaron, 2005, p. 292; Arumí and Vargas-Urpi, 2018, p. 431). Generally speaking, these additions perform the functions of recovering missing information, asking for clarification, coordinating turn-taking, and seeking confirmation. However, the examples presented below are the types we would not expect to appear in formal settings like interpreter-mediated political press conference. These include not only some text-oriented ones, produced to comment on translation and to clarify something for the audience, but also interaction-oriented ones, which can serve as invitation for journalists to self-translate questions.

#### Comment on Translation

Example 1 depicts a scenario where the Director was asked by a journalist to pick a Michael Jackson song which, according to him, would give an auspicious sign for the future US-Taiwan economic relationship. The Director responded that he was actually not a fan of Michael Jackson and he mentioned his kids who knew better about modern music than him. But according to the Director, even his kids were not followers of Michael Jackson. Instead, he listed three music bands that were particularly popular among his children's generation. However, these band names are culturally loaded proper names that fell outside the interpreter's knowledge of contemporary pop-culture. Similar to the situation described by Meyer (2008), the interpreter at first tried to reproduce the English names with “phonetic adaptations” (p. 113). However, she soon gave up and produced an extra-textual addition, explaining that her failure to interpret the proper names was due to her age and lack of shared knowledge with the speaker. An utterance like this may reflect the interpreter's attempt to save face for “self-preservation” (Monacelli, 2009), in regard to the presence of some bilingual journalists who can monitor her interpreting performance.

**Table:** 

**Example 1**		
**Source text: English**	**Target text: Chinese**	**Back translation**
Director: See, my kids are educating me about more modern music, but they weren't around for the Michael Jackson years. They are groups like Gogol Bordello or Cake or Kings of Leon, but we skipped the Michael Jackson years.	I: 我的小孩等到他們夠大來聽那些通俗音樂的時候，已經跳過 Michael Jackson 那個年代了。他們聽的是 [Gordon Boblian], Cake… *不好意思，我的年紀太大了，所以這些也不是我知道的。*	My kids, when they were old enough to listen to popular music, the Michael Jackson era had already passed them. The music they listened to was [Gordon Boblian], Cake… Sorry, I'm too old, so these (names) are not what I know.

#### Clarification for the Audience

The following two extra-textual additions (underlined in the back translation) are interpreters' attempts to clarify the speaker's meaning to the audience by providing them with more relevant information about the Director's utterances.

By producing such additions, the interpreter assumed the role of a “cultural mediator” who “facilitates communication, understanding, and action between persons or groups who differ with respect to language and culture” (Katan, 2004, p. 17). In Example 2, the interpreter produced an addition to help the audience realize why time passing made the Director feel that it was difficult to carry his family name “Young.” The reason lies in that the English adjective “young” corresponds to 年輕 (*nian qing*) in Chinese. This extra-textual addition is marked by a shift in personal deixis from first person to third person before the interpreter finally returned to the first-person voice.

**Table:** 

**Example 2**		
**Source text: English**	**Target text: Chinese**	**Back translation**
Director: God gives everybody a fate, doesn't He? And in my case, He gave me a name that is more and more difficult for me to carry as I grow older.	I: 各個人的命運各自不同。因為我的姓，就是他的英文名字的姓“年輕”, 所以讓我覺得年紀越大的話越難以承受。	Everybody has their own destiny. Because of my name, his English surname is [*nian qing*] (Mandarin for “Young”), it makes me feel more and more difficult to carry as I grow old.

**Table:** 

**Example 3**		
**Source text: English**	**Target text: Chinese**	**Back translation**
Journalist: And, secondly, my second question is what is the timing, purpose, meaning, and issues of the possible visit of the Veteran Minister Eric Shinseki next year? Thank you.	I: 第二個問題要請教的就是說，據聞呢，美國的叫作退休官兵部,退伍軍人部, 類似台灣的那個退役服軍人部的部長叫 Shinseki, 據說已經有人向他邀請來台灣訪問了, 那請問他訪問的時間, 訪問的目的, 還有他的訪問 的意義如何？	[My second question is: It is heard that the Ministry of retired military officers and soldiers of the US, the *one* like the Ministry of Veterans' Affairs in Taiwan, its Minister is Shinseki. It is said that he has been invited to visit Taiwan. So, I'd like to know the timing, purpose, and meaning of his visit?]

In Example 3, it is evident that the interpreter tried to locate a term corresponding to the foreign institutional title “Veteran Minister” in the target cultural system. For such a purpose, she initiated an extra-textual addition to broker the culturally relevant information to the audience. Despite the impression that the interpreter thus becomes a “principal” in Goffman's (1981) terms, the addition shown here is reminiscent of Wadensjö (1998) suggestion that “interpreters cannot avoid functioning as intercultural mediators through their translation activity” (p. 75).

#### Invitation to Translate

Five instances of extra-textual additions, all produced by one interpreter in one session, are interaction-oriented, showing that the interpreter invited the journalist to translate the question that had just been posed.

These extra-textual additions, as exemplified in Example 4 above, are particularly interesting to note, as they have not been reported elsewhere before. Instead of associating these additions with reduced professional standards, we would like to draw on Inghilleri (2005) point of the interpreting profession being a “zone of uncertainty” to view the given phenomenon. The notion of uncertainty, on the one hand, leaves the interpreters socially “vulnerable to exercises of power outside of their control” (p. 81), but, on the other, indicates that they have the potential to “challenge the normative practices specific to their own or others' professions” and to “contribute to the production of a different social/interactional order” (p. 75–76). No definite knowledge can be gained from the corpus data alone in relation to the cause of the observed addition, which may have been induced by the interpreter's fatigue, inefficient note-taking, or simply a wish for interaction. However, we can argue that, by sending a translation request to the bilingual journalists, the interpreter creates new discursive and social practices, which then shed new light on her role.

**Table:** 

**Example 4**		
**Source text: English**	**Target text: Chinese**	**Back translation**
Journalist: I have a question about arms sales. …	I: *這位朋友您的問題要不要自己翻譯？*?	this friend, do you need to translate your question by yourself?

### A2: Connective Additions

**Figure 2:**
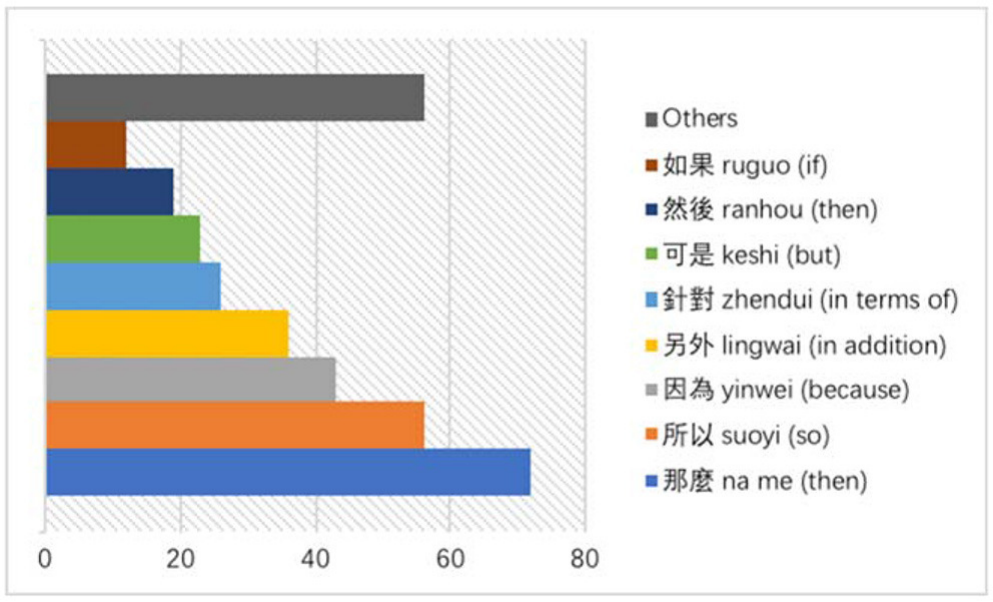
Frequency of connectives added by interpreters.

Altogether, 343 instances of connectives were identified as interpreters' additions. On a normalized basis of 10,000 tokens, there are 119.2 instances in the Chinese TT. Figure 2, below, shows the top eight most added connectives in the AIT Interpreting Corpus.

The observed frequencies for these connectives, from the highest to lowest, are “那麼” *na me* (then), “所以” *suoyi* (so), “因為” *yinwei* (because), “另外” *lingwai* (in addition), “針對” *zhendui* (in terms of), “可是” *keshi* (but), “然後” *ranhou* (then) and ““如果” *ruguo* (if). As has been stated earlier, these connectives can easily be “grammaticalized” into discourse markers for creating discourse coherence, rather than simply functioning to link interclausal relations.

It is worth noting that our observation regarding connective additions in interpreted Chinese contradicts the claim made by Chinese grammarians (Wang, 1984; Lian, 2010) that Chinese, as a highly paratactic language, is not necessarily dependent on connectives to maintain coherence once sufficient contextual information is provided. Rather, our findings suggest that extra connectives are added in interpreting, which have no corresponding markers in the source English texts. In the absence of definite knowledge regarding the implicit meaning the source speaker intended to convey, we chose to discuss the contribution of the added connectives regarding their linguistic function *per se*, rather than categorically relate them to evidence of explicitations.

In Example 5, the speaker offered advice to the governing party of Taiwan on how to cooperate with the opposition, which came down to three aspects that were introduced following the exemplifying marker “like” in three consecutive phrases. As can be seen, the interpreter framed the Director's three pieces of advice in an orderly sequence using Chinese: “第一點” *diyidian* (first), “第二點” *dierdian* (second), and “第三點” *disandian* (third). Additions of these connectives, or more precisely, frame markers, echo the findings in Fu (2017), who suggested that they are like “handrails in order for the discourse to be hierarchically or orderly structured” (p. 853). Because of these added linguistic elements, the interpreted discourse becomes clearly organized, as if the interpreter is signposting for the audience the ideas unfolding within the speaker's thought processes. Therefore, it seems that the interpreters are inclined to add connectives, irrespective of the language direction (English↔Chinese) in which they are required to work.

**Table:** 

**Example 5**		
**Source text: English**	**Target text: Chinese**	**Back translation**
Director: In that sense, we encourage the pursuit of dialogue between the government here and opposition on important issues like the strengthening of democracy, respect for differing opinions, and the future direction of cross-Strait relations.	I: 因此我們也鼓勵目前台灣的政府也跟在野黨有更多的互動和交流[…]針對以下的項目,第*一點*就是如何去鞏固我們民主的基礎；第*二點*,如何去尊重各種不同的意見；第*三點*,針對未來兩岸關係要如何發展,要有更多的一些磋商。	So, we also encourage the current Taiwan government to have more exchanges and communication with the opposition party on the following issues: *F*irst, how to strengthen the base of our diplomacy? *Second*, how to respect different opinions? Third, as for the future direction of cross-Strait relations, it needs more exchanges of views.

In fact, as suggested by Mason and Hatim (2002), for ease of audience understanding, “effective consecutive output thus exhibits a clear outline of the way a text is structured” (p. 262). In Example 6, the interpreter added a range of connectives that function mostly as DMs encompassing a series of related events and actions under one larger discourse frame. The use of such connectives renders the interpreted Chinese more signposted and structured with cohesive links. Overall, the added connectives help to serve the given communicative purpose. By connecting the clausal relations between various discourse units, these connectives help to provide almost indisputable grounds for the Director's opposition against the attempt on the part of Taiwan to revise the Taiwan Relations Act.

**Table:** 

**Example 6**		
**Source text: English**	**Target text: Chinese**	**Back translation**
Director: The US Constitution is over 200 years old and we still abide by that. No one in America has called for its revision or abrogation. It's stood us pretty well. I would also note that the principal communiqués governing our relationship with the Mainland—two of them anyway—are older than the Taiwan Relations Act. The Mainland, I don't think, has called for a revision of those either. I think the document has served us pretty well as a guideline to our policy. I don't think the age of the document, if the fundamental principles in it are correct, makes a difference.	I: 在美國我們的憲法也已經超過200多年了, 大家也是持續在遵循, 並沒有人說憲法應該修改, 所以 200多年來一直屹立不搖。 那麼, 針對跟中國還有台灣的關係, 除了《台灣關係法》之外, 還有另外兩個公報, 其實比《台灣關係法》簽訂的時間更早, 可是也沒有聽到中國說要修訂那些公報等等。因為像一份法律, 像《台灣關係法》這樣的法律, 它主要是反映美國對台灣政策的一個重要綱領, 並不會因時代久遠, 而失去它的意義。	[In America, our constitution has been passed over 200 years too. People still abide by it and no one has called for its revision. So, it has stood rock-solid for over 200 years. Then, regarding our relations with China and with Taiwan, apart from the Taiwan Relations Act, there are two additional communiqués which were actually signed earlier. But I didn't hear from China to ask for revision of those communiqués. Because an act like TRA, it serves mainly as an important guideline reflecting the American policies toward Taiwan, it will not lose meaning because of time.]

### A3: Emphasizing Addition

**Figure 3:**
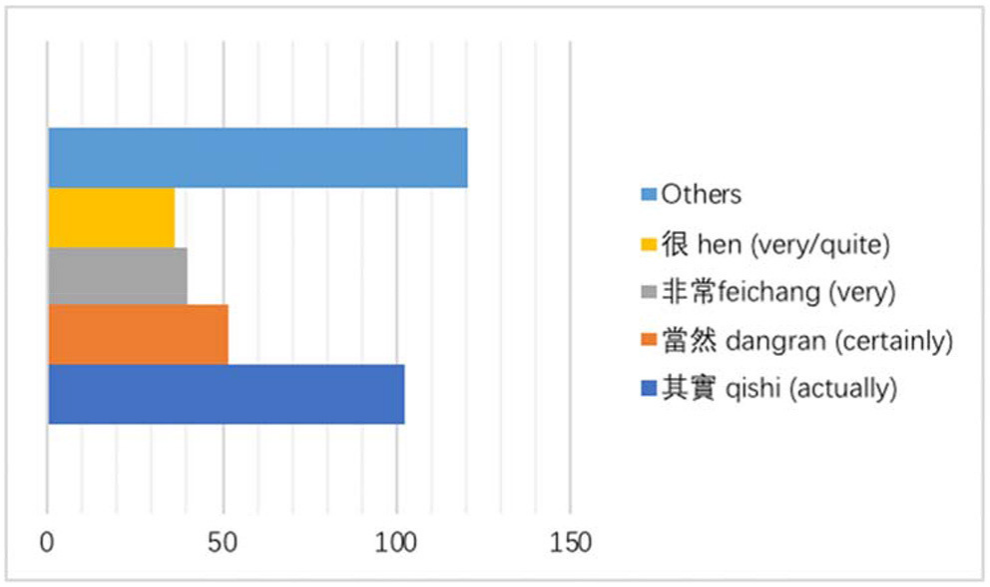
Frequency of intensifiers added by interpreters

The interpreters' tendency to add emphasizing additions in the form of intensifiers is also remarkable, which can be shown by their total observed frequency (350) or normalized frequency (121.6) on a basis of 10,000 tokens in the interpreted Chinese. Figure 3 provides the frequencies of occurrence of the top four most added intensifiers, i.e., “其實” *qishi* (actually) “當然” *dangran* (certainly) “非常” *feichang* (very) “很” *hen* (very/quite).

Among these intensifiers, “其實” *qishi* accounts for about 29% of all the identified emphasizing additions. The emphasis on “其實” *qishi* is that it has evolved into a discourse maker, which can not only achieve an intensifying function toward the speaker's conveyed propositional attitude but can signal coherent relations between discourse units (Wang et al., 2010). The following examples reveal the linguistic functions and the ensuing implications of these added linguistic devices.

Example 7, below, gives two additional stretches of source text where emphasizing additions are littered through the target interpretation.

**Table:** 

**Example 7**		
**Source text: English**	**Target text: Chinese**	**Back translation**
**(7a)**		
Director: I continue to believe that US–Taiwan ties are historic and durable.	I: 我一直持續地相信美國和台灣之間的關係是非常具有歷史性，而且會非常的持久。	I always continue to believe that US–Taiwan ties are very historic and very durable.
**(7b)**		
Director: At the same time, we continue to see the Mainland's rapid buildup across the Strait as a force for instability and a threat to the status quo, and we raise this in our discussions regularly with Beijing.	I: 同時我們也看到中國它不斷的武力擴張，軍事方面的發展，這個對兩岸關係造成非常不利的影響。對此我們也常常跟中方提出我們的意見。	At the same time, we also see China, its relentless military expansion, military development. This has inflicted a very bad impact on the cross-Strait relations. We raise this in our discussions regularly with China.

In Example (7a), the interpreter has added two intensifiers, “非常” *feichang* (very), before the adjectives “historic” and “durable” that modify “US-Taiwan relations,” thus reinforcing the semantic strength conveyed in the source statement. Likewise, an intensifier “一直” *yizhi* (always) added before “*continue to believe*” contributes to the same emphatic effect with respect to the speaker's certainty and confidence in the promise of US-Taiwan relations.

However, it is interesting to note that in Example (7b), when the statement is addressed toward mainland China, the added intensifier *feichang* also intensifies the perceived negativity toward this subject. Admittedly, the interpreter's rendition compresses (Bartlomiejczyk, 2006) or perhaps even distorts the Director's evaluation of mainland China, from a specific “*force for instability and a threat*” to a rather general “*bad impact*.” But with the original nominal phrases rendered into a verbal predicate “*inflicted*” (*a bad impact*), this assumed threat from mainland China to Taiwan is also made more concrete and tangible, as if it was something that had already happened. On this point, the added *feichang* is not meaningless, given that the absence of it would not make the interpreted utterance unnatural but only make the intensity level lost.

Example 8, again, offers more than one case of source-target interpretation emphasizing additions.

**Table:** 

**Example 8**		
**Source text: English**	**Target text: Chinese**	**Back translation**
**(8a)**		
Director: We have a good framework in place for managing our security relationship with Taiwan under the Taiwan Relations Act, and it has now been effective for over 30 years.	I: 其實針對台灣的安全如何維護，過去30年來，我們都是在《台灣關係法》的架構之下進行的，而且30年來其實進行的都很穩健。	Actually, in terms of our security relationship with Taiwan, in the past 30 years, we have been operating under the framework of TRA, and it has been actually very effective for 30 years.
**(8b)**		
Director: But we're also aware of the relentless buildup of military strength by the PRC, much of it directed precisely toward Taiwan.	I: 但是我們也了解在中國，他們其實是不斷地是在提升他們本身的軍事實力，也包括一些在海峽的對岸有部署更多針對台灣的飛彈。	But we're also aware that China, they are actually continuously enhancing their military strength, which also includes deploying more missiles toward Taiwan across the Strait.

When the statement is addressed toward Taiwan in Example (8a), the interpreter has added “其實” *qishi* (actually) and “很” *hen* (very) before “effective,” which contributes to the sense of even closer ties between the US and Taiwan. This is because the semantic force suggestive of how the Taiwan Relations Act has been operating successfully is enhanced.

However, when the statement is addressed toward mainland China, the interpreter's behavior is consistent with that observed in Example (7b). As shown in Example (8b), the interpreter added an intensifier *qishi*, together with rendering the nominal “*buildup of military strength*” into the verbal “*enhancing military strength*.” A closer examination finds that the use of *qishi* functions as DMs, signaling various relationships, such as contrast or commentary, between the upcoming proposition and previous assumptions, which helps to reflect the speaker's commitment to the truth of his proposition.

According to Hyland (2005), intensifiers can “emphasize certainty and construct rapport by marking involvement with the topic and solidarity with an audience, taking a joint position against other voices” (p. 53). On this basis, due to the added intensifiers, the Taiwanese audience would be exposed to no alternative viewpoints other than the certainty about the commitment of the US to Taiwan's defense and the certainty about the imminent threat of mainland China to cross-Strait relations.

## Discussion

The observed additions are now discussed in terms of their linguistic functions. In doing so, attention is given to how the uniqueness of the interpreted event at the AIT (American Institute in Taiwan) can contribute to interpreters' extra-textual additions and with what implications for the interpreted discourse due to their added connectives and intensifiers, as grammaticalized into DMs.

First, a mere 6% of identified extra-textual additions are used in interpreting with a range of functions, such as recovering missing information, asking for clarification, coordinating turn-taking, and seeking confirmation. There are, in addition, also others, which are produced to comment on translation, clarify information for the audience and invite journalists for self-translation. Compared with the former types that have already been validated by some interpreting codes of ethics (e.g., NAJIT, 2016), the latter are rather context-specific in that they are not expected in high-profile and rigidly structured press conference interpreting. As emphasized by Downie (2017), “it is very difficult to talk of any inflexible standards for interpreting that are equally valid across all forms and contexts” (p. 266). So, rather than judging these additions by any preconceived professional standards, we decided to look at them in relation to the special variable within the given interpreted event.

In this regard, one significant feature differentiating interpreting at the AIT from interpreting at other conference settings, such as mainland China's CPPC interpreting, is that the AIT Directors would make direct interaction with on-site journalists or toward interpreters from time to time. Such interactions are detectable in the audio-video recordings between the Directors and journalists regarding topics such as Taiwan's weather reports, American baseball games, or their hobbies in Taiwan, leading to some spontaneous laughter at the auditorium. Or, they could interact occasionally with the interpreters by explaining to them some culture-specific items which might be difficult to interpret. It is interesting to note that the Directors with proficient Chinese speaking skills could make the interactions either in Chinese or by code-switching between Chinese and English. In this case, we assume that the interpreters' extra-textual additions, notably those interaction-oriented ones, are motivated by the interactions from the Director. Considering the AIT was established to facilitate the informal but strong relations between Taiwan and the US, as stipulated in the Taiwan Relations Act, the Directors' interactions are indeed oriented toward such social and communicative goals. This uniqueness underlying interpreting at the AIT may therefore contribute to a greater “visibility” of interpreters, which is manifested through their production of extra-textual additions.

Second, with respect to the added connectives and intensifiers, they account for almost 94% of all the identified additions, and given their grammaticalization process into discourse markers, such a great proportion of additions can then have a bearing on the interpreted discourse. It is known that DMs are like “the oil which helps us perform the complex task of spontaneous speech production and interaction smoothly and efficiently” (Crystal, 1988, p. 48). So, these added linguistic devices can contribute to marking continuation of ideas in discourse flow.

Of particular note is that DMs are not language-specific but prevalently used in both spoken English and Chinese to create discourse coherence (Traugott, 1995; Fraser, 1999; Traugott and Dasher, 2002; Wang and Huang, 2006; Wang et al., 2010). This is why an extra number of connectives and intensifiers have indeed been found in interpreting from English to Chinese despite some Chinese grammarians' claim that Chinese language is not dependent on such grammatical items to maintain coherence (Wang, 1984; Lian, 2010). In fact, the proliferated additions of connectives and intensifiers can reflect interpreters' competence in monitoring, organizing and managing the interpreting output. Just as rightfully suggested by Shlesinger (1995), the success of interpreting “will depend on whether target text recipients can achieve second-degree interpretation with minimal extra processing effort” (p. 209). Specifically, it is undeniable that the added connectives, such as 因為 *yinwei* (because), 所以 *suoyi* (so), 然後 *ranhou* (then), 可是 *keshi* (but), etc. have helped render the target Chinese more coherent at the discourse level. However, the added intensifiers such as 其實 *qishi* (actually) and 事實上 *shishishang* (in fact) should deserve special mention. This is because apart from signaling coherent relations between discourse units, these emphasizing additions have also intensified the original authorial attitudes, notably those assuming that the mainland constitutes a threat to Taiwan. Given the decades of discord across the Taiwan Strait, such rhetorical effects ensuing from interpreting are not insignificant but worth more scholarly attention in the future.

## Conclusion

This study has investigated additions in a rarely visited setting, interpreter-mediated press conferences for the American Institute of Taiwan (AIT). The examples used in this study feature an under-explored language-pair direction (i.e., interpreting from English into Chinese) in response to the overwhelming amount of research focusing on the Chinese Premier's Press Conference interpreting which is conducted in the opposite direction. Three types of additions made by professional Taiwanese interpreters were identified and examined closely. Firstly, extra-textual additions were observed not merely in community-based settings, but also in professional press conferences. In addition, the repeated additions of connectives and intensifiers that were observed contradicts the traditional thought that Chinese is not dependent on these devices to maintain coherence. It has also been found that interpreters' interaction-oriented extra-textual additions are somewhat motivated by the Director's interactions with on-site journalists, and that their added connectives and intensifiers are mostly a result of the grammaticalization of such linguistic devices into Discourse Markers occurring prevalently in both English and Chinese. Despite the findings of the current study, more research using other research methods, including the use of surveys and interviews with interpreters could help uncover more about the precise nature and trends evident during such interpreting activities.

## Data Availability Statement

The original contributions presented in the study are included in the article/supplementary material, further inquiries can be directed to the corresponding author.

## Author Contributions

RL performed data analysis and wrote the first draft of the manuscript. AC contributed to data-coding and interpretation of results. KL contributed to revising the manuscript and giving additional insights. All authors approved the final version of the manuscript for submission.

## Conflict of Interest

The authors declare that the research was conducted in the absence of any commercial or financial relationships that could be construed as a potential conflict of interest.

## Publisher's Note

All claims expressed in this article are solely those of the authors and do not necessarily represent those of their affiliated organizations, or those of the publisher, the editors and the reviewers. Any product that may be evaluated in this article, or claim that may be made by its manufacturer, is not guaranteed or endorsed by the publisher.
